# Memory-Efficient Synaptic Connectivity for Spike-Timing- Dependent Plasticity

**DOI:** 10.3389/fnins.2019.00357

**Published:** 2019-04-24

**Authors:** Bruno U. Pedroni, Siddharth Joshi, Stephen R. Deiss, Sadique Sheik, Georgios Detorakis, Somnath Paul, Charles Augustine, Emre O. Neftci, Gert Cauwenberghs

**Affiliations:** ^1^Integrated Systems Neuroengineering Laboratory, Department of Bioengineering, University of California, San Diego, La Jolla, CA, United States; ^2^Department of Computer Science and Engineering, University of Notre Dame, Notre Dame, IN, United States; ^3^aiCTX, Zurich, Switzerland; ^4^Department of Cognitive Sciences, University of California, Irvine, Irvine, CA, United States; ^5^Intel Corporation - Circuit Research Lab, Hillsboro, OR, United States

**Keywords:** synaptic plasticity, neuromorphic computing, data structure, memory architecture, crossbar array

## Abstract

Spike-Timing-Dependent Plasticity (STDP) is a bio-inspired local incremental weight update rule commonly used for online learning in spike-based neuromorphic systems. In STDP, the intensity of long-term potentiation and depression in synaptic efficacy (weight) between neurons is expressed as a function of the relative timing between pre- and post-synaptic action potentials (spikes), while the polarity of change is dependent on the order (causality) of the spikes. Online STDP weight updates for causal and acausal relative spike times are activated at the onset of post- and pre-synaptic spike events, respectively, implying access to synaptic connectivity both in forward (pre-to-post) and reverse (post-to-pre) directions. Here we study the impact of different arrangements of synaptic connectivity tables on weight storage and STDP updates for large-scale neuromorphic systems. We analyze the memory efficiency for varying degrees of density in synaptic connectivity, ranging from crossbar arrays for full connectivity to pointer-based lookup for sparse connectivity. The study includes comparison of storage and access costs and efficiencies for each memory arrangement, along with a trade-off analysis of the benefits of each data structure depending on application requirements and budget. Finally, we present an alternative formulation of STDP via a delayed causal update mechanism that permits efficient weight access, requiring no more than forward connectivity lookup. We show functional equivalence of the delayed causal updates to the original STDP formulation, with substantial savings in storage and access costs and efficiencies for networks with sparse synaptic connectivity as typically encountered in large-scale models in computational neuroscience.

## 1. Introduction

Extensive research in the field of artificial neural networks (ANNs) in the past decade has given rise to diverse neuron functions, network topologies, and training techniques (Nair and Hinton, 2010; Krizhevsky et al., 2012; Goodfellow et al., 2014; Kingma and Ba, 2014; Ioffe and Szegedy, 2015), capable of solving complex cognitive tasks, such as image classification (Krizhevsky et al., 2012), sequence generation (Graves, 2013), speech recognition (Graves et al., 2013), and game playing (Silver et al., 2016). However, the components of these algorithms are normally only loosely based on actual biological neural networks, particularly with respect to the non-local learning rules (e.g., the widely used backpropagation algorithm, Rumelhart et al., 1986) and the continuous activation functions (e.g., sigmoid unit and rectified linear unit). Spiking neural networks (SNNs), in contrast, incorporate multiple aspects of biological nervous systems into its components (Gerstner and Kistler, 2002), including biologically relevant neuron models, binary activation functions and communication, event-driven processing, and local learning rules (i.e., where all the information required for adjusting parameters between neurons is collocated with these neurons). The neuron models can range from simple single-variable differential equations (e.g., McCulloch-Pitts and integrate-and-fire), to complex systems with dynamics more homologous to real neurons (e.g., Hodgkin-Huxley). In SNNs, neurons communicate between each other via a binary event known as an action potential (or *spike*), which is elicited whenever a neuron variable (typically, the membrane potential) crosses a threshold value. Whenever a neuron produces an action potential, this spike event information is conveyed to its population of downstream post-synaptic neurons, resulting in an update of their respective internal variables based on the values of synaptic efficacy (or *weight*). Due to their binary nature, the time at which spikes occur is essential information when training SNNs.

The origins of hardware designed to emulate the biological nervous system, also known as neuromorphic systems Mead (1990), targeted design of neural properties at the device level, with natural focus on analog circuits (Maher et al., 1989; Andreou et al., 1995; Koch and Mathur, 1996). More recently, however, neuromorphic systems such as TrueNorth (Merolla et al., 2014), SpiNNaker (Furber et al., 2014), and Loihi (Davies et al., 2018) were designed with purely digital components, being capable of emulating large-scale SNNs with real-time dynamics in the millisecond timescale. Additionally, large digital systems have the advantage of being more readily verifiable in simulation and a software-hardware equivalence is typically possible. While ANNs operate in a sequential manner, where data propagates through the network one layer at a time, neuromorphic systems typically present multiple cores running in parallel at biological timescales, with synaptic memory local to each core. Systems with distributed processing and memory move away from the traditional von Neumann architecture, where memory is centralized and a high-frequency global clock is responsible for fast computation and memory access (Merolla et al., 2014).

Among the bio-inspired learning mechanisms, spike-timing-dependent plasticity (STDP) is perhaps the most widely considered form of induced synaptic modification (Markram et al., 1997). STDP originated from experimental data collected in cultures of dissociated rat hippocampal neurons, where scientists observed that a causal relationship between spike times of pre- and post-synaptic neurons could induce synaptic strengthening or weakening, and this change was correlated with the relative temporal difference of spikes (Bi and Poo, 1998). The experiments showed that long-term potentiation and long-term depression could both be induced in synapses depending on the order of spike occurrence, where a causal relationship (i.e., pre-synaptic neuron spikes before post-synaptic neuron) potentiated the synapse, while an acausal relationship (i.e., post-synaptic spikes before pre-synaptic) weakened the synapse. The authors then approximated the measured synaptic modification with a mathematical model. In the model, the STDP function (or *kernel*) defines the change of the weight as a function of the relative time between pre- and post-synaptic action potentials, and the duration of the causal (and acausal) influence of spikes is called the STDP learning window (Sjöström and Gerstner, 2010). An important aspect of STDP is that, though it is a local learning rule, weight updates occur at the onset of both pre- and post-synaptic spikes, requiring for the algorithm to be able to not only identify all neurons which the pre-synaptic neuron sends its spikes to, but also locate all the neurons which the post-synaptic neuron receives its spikes from. This is a fundamental property of STDP, and throughout our work we will refer to reading the neuron addresses and weights from pre-to-post connectivity as *forward access* and reading from post-to-pre connectivity as *reverse access*.

In traditional ANNs, the typical data structure used to represent the weights between neurons is a dense matrix, constituting a fully connected topology. However, more realistic and biologically relevant neural networks, such as small-world and locally connected random networks (Bassett and Bullmore, 2006; Bullmore and Sporns, 2009; Seeman et al., 2018), do not conform to this structured topology. In these cases, synaptic weight storage costs can benefit greatly using compressed representations. For physical realizations of the STDP learning rule, the arrangement used to organize the synaptic weights in memory has a direct impact on the ease of forward and reverse access. As we will later show, dense matrices typically have the advantage of natively facilitating both types of connectivity access. Conversely, compressed memory arrangements suffer greatly when trying to access in the reverse direction, making causal STDP weight updates in these structures computationally intensive. In this work, we discuss the complexity of storing and accessing synaptic weights in different types of data structures and their impact on implementations of the STDP algorithm, and propose a novel method of performing STDP using only single-direction connectivity access, consequently taking advantage of compressed structures.

Storage costs associated to synaptic weight memory arrangements have been previously studied (Moradi et al., 2013; Pedroni et al., 2016; Joshi et al., 2017; Kornijcuk et al., 2018). In Materials and Methods, we give an overview of four typical data structures used for representing synaptic weights, and analyze storage costs based on different network parameters (number of neurons and weight bit-length) and varying degrees of network connectivity density. We extend our analysis to verify the memory access cost and efficiency associated to each data structure, focusing particularly on the computational complexity and requirements for performing STDP. Inspired by our previous work (Pedroni et al., 2016), we propose a definite pre-synaptic-driven solution for obtaining a quantitatively equivalent algorithm to STDP. Previous attempts in approximating STDP using forward-only connectivity include (1) simplifying the STDP rule by equally updating all the synaptic weights based on recent spike activity (Bichler et al., 2012; Yousefzadeh et al., 2017), (2) using other variables (usually post-synaptic membrane potential) as a proxy for the post-synaptic spike times when computing causal updates (Brader et al., 2007; Davies et al., 2012; Lagorce et al., 2015; Sheik et al., 2016), and (3) delaying the weight updates (Jin et al., 2010; Davies et al., 2018). In the discussion, we compare our method to these, particularly with the third type, currently present in SpiNNaker and Loihi, and explain how our solution can produce exact STDP while previous methods rely on particular balanced firing rate conditions in the network or simply produce qualitative approximations to STDP. In Results, a network composed of 256 pre-synaptic and 256 post-synaptic neurons is simulated using our proposed method and compared against the original STDP learning rule, showing that our method produces the same post-synaptic membrane potentials, resulting in identical spiking activity and synaptic weights.

## 2. Materials and Methods

### 2.1. Digital Neuromorphic Core

Neuromorphic systems emulate the biophysics of neural computation in correspondingly tailored electronic circuits (Mead, 1990). Whereas artificial neural networks are typically deployed as software applications in general purpose hardware, neuromorphic systems are normally developed accounting for the properties and limitations that a physical hardware implementation entails. These include biologically plausible neurons (i.e., spiking neurons) and learning rules, binary event communication (i.e., neurons communicating via spikes), limited and local synaptic memory, and parallel and distributed neuron processing (Mahowald, 1993; Liu and Delbruck, 2010; Indiveri et al., 2011; Park et al., 2017).

**Figure 1:**
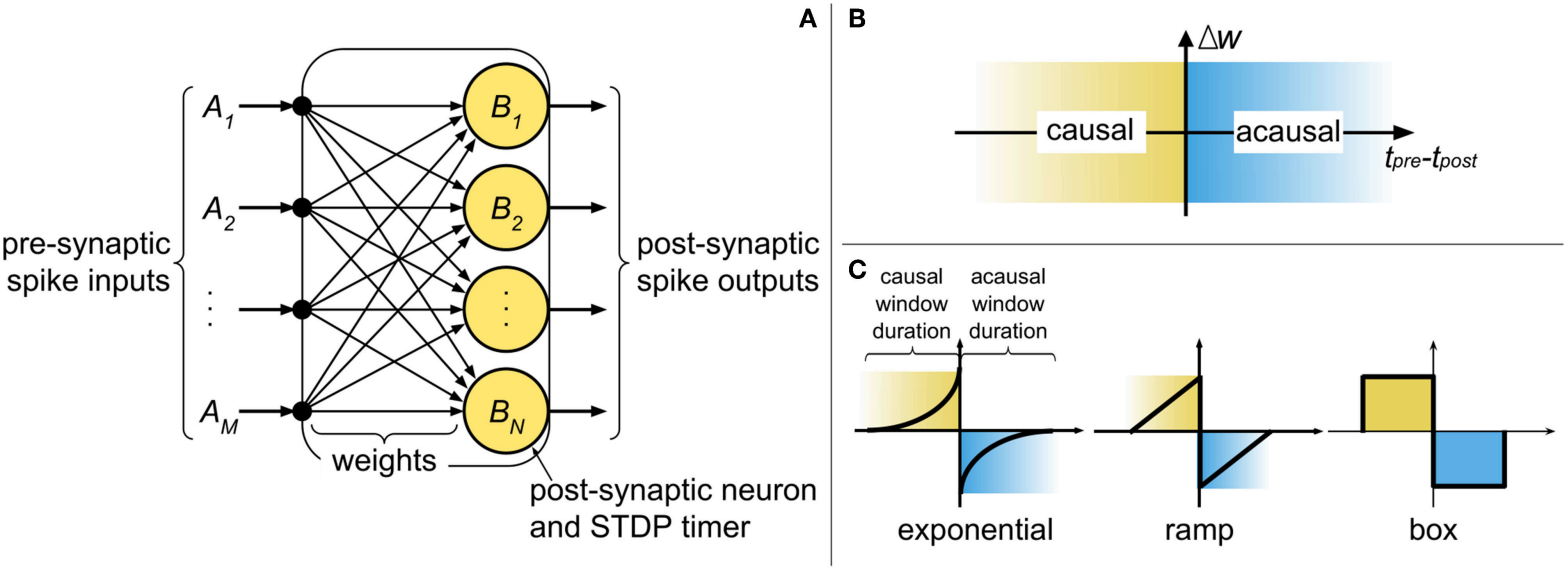
**(A)** An abstract representation of a neuromorphic spiking neural network core and the components required for implementing pre-synaptic spike-driven STDP. **(B)** The causal and acausal regions of the STDP function. **(C)** Typical STDP kernels implemented in neuromorphic systems.

The current state-of-the-art digital neuromorphic processors, such as TrueNorth (Merolla et al., 2014) and Loihi (Davies et al., 2018), partition the network into *cores*, where typically the population of post-synaptic neurons in a core shares inputs from a common pool of pre-synaptic neurons. At a high level, the core comprises of a digital finite-state machine, with weights stored in digital memory elements (e.g., random access memory - RAM), and with the state of the neural and synaptic variables progressing in discrete time steps (Δ*t*), representing the temporal precision of the system. Figure 1A illustrates an abstract digital neuromorphic core and its components. The core operates by processing incoming pre-synaptic spikes (irrespective of their origins) and updating the post-synaptic state variables (e.g., membrane potential) with the associated weight between the pre- and post-synaptic neurons. Once all pre-synaptic spikes have been processed, the post-synaptic neurons are evaluated. Any new post-synaptic spike is then routed to its destination (on another or the same core), where there it is treated as an incoming pre-synaptic spike and is buffered to be used in the next system time step.

For realizing STDP learning in digital neuromorphic systems, a core must locally store (or have access to) the following: pre-synaptic spike times, synaptic weights, and post-synaptic neurons and spike times. Collocating the synaptic weights with the post-synaptic neurons ensures that all the information required for local and distributed learning strategies can be accessed with minimum overhead (Joshi et al., 2017). Interestingly, since our proposed method operates in pre-synaptic spike-driven fashion, a core does not require storing the pre-synaptic spike times. In other words, the spike times only need to be stored at the origin of the spike (i.e., at the post-synaptic neuron).

Lastly, an important consideration throughout our work is that we analyze the storage and access efficiency of the different memory arrangements based on the data structure used for storing synaptic weights. For this, we abstract away the physical storage elements by considering that each position in memory contains only a single “packet” of information (of arbitrary length), and that only one position in memory can be accessed at a time (i.e., each read/write command targets one “packet” at a time). Though memory storage and access in dynamic RAMs (DRAMs), for example, is typically not performed on an arbitrary number of bits (i.e., usually each read/write command targets a few bytes at a time), and complete random access is less efficient than bursts of sequential addresses of data, understanding the efficiency of each memory arrangement would become too involved if we were to consider the intricacies of exact physical models. For simplicity, we consider that *storage costs* take into account only the total number of bits for storing the connectivity and weight tables, and that each read/write command accesses only one address of the table at a time. Thus, the computational complexity of locating neuron addresses and weights in the data structures, denoted as *access cost*, considers the number of variables which must be accessed until the desired information is located, and can perhaps serve as a proxy for indirectly evaluating latency and energy of the methods.

### 2.2. Spike-Timing-Dependent Plasticity (STDP)

Spike-Timing-Dependent Plasticity is a biologically inspired form of Hebbian learning which considers the relative spike time of pre- and post-synaptic neurons for updating the synaptic efficacy (or weight) (Caporale and Dan, 2008). Though STDP is believed to be a fundamental learning mechanism in the mammalian brain (Dan and Poo, 2004) and has been widely explored in computational neuroscience (Song and Abbott, 2001; Izhikevich, 2007; Sjöström and Gerstner, 2010), results obtained in machine learning applications (Nessler et al., 2009; Diehl and Cook, 2015; Yousefzadeh et al., 2017; Kheradpisheh et al., 2018) suggest it may also be an interesting solution in non-biological scenarios.

STDP operates by modifying synaptic weights at the onset of pre- and post-synaptic spikes. “Causal updates” occur when a pre-synaptic spike precedes a post-synaptic spike, resulting in an increase in synaptic efficacy (i.e., long-term potentiation). Conversely, when a pre-synaptic spike proceeds a post-synaptic spike, an “acausal update” occurs and the efficacy is reduced (i.e., long-term depression). Figure 1B identifies the causal and acausal regions of the STDP function. The strength in which these changes take place is dependent on the temporal difference between the spikes, and can also consider other factors (such as the current weight value). In sum, the polarity of change depends on the order of the spikes, while the intensity of change depends on the temporal difference of the spikes. The basic model for STDP is defined mathematically by

(1) $$\begin{array}{r} \Delta w_{ij}=\overset{T_{j}}{\underset{a=1}{\sum }}\overset{T_{i}}{\underset{b=1}{\sum }}W\left(t_{j}^{a}-t_{i}^{b}\right), \end{array}$$

where the weight change between pre-synaptic neuron *j* and post-synaptic neuron *i* is defined by the STDP kernel, *W*, using all *T*_*j*_ pre-synaptic spike times, *t*_*j*_, and all *T*_*i*_ post-synaptic spike times, *t*_*i*_.

The STDP kernel is a function which defines how weights are modified based on the relative temporal difference between pre- and post-synaptic spikes. Figure 1C highlights the causal (when *t*_*pre*_ < *t*_*post*_) and acausal (when *t*_*pre*_ > *t*_*post*_) regions of the STDP function in three commonly used kernels: (truncated) exponential, ramp, and box. The basic STDP model in Equation (1) considers a causal relationship of infinite duration between all pre- and post-synaptic spikes. However, physical realizations of STDP cannot account for a limitless amount of data to be stored and analyzed at every instant of weight update. Therefore, two considerations must be made for temporal spike interaction when implementing STDP in a neuromorphic system: (1) the duration of the kernel is finite and (2) the number of spike times which can be stored is finite. For the first consideration, the typical STDP kernels in Figure 1C present finite causal and acausal window duration. In hardware, this duration is defined by the limit of the STDP timers used in the system. The exponential kernel, in theory, has a window duration of infinite time; nonetheless, for physical realizations of the kernel, we define a limit (i.e., truncation) on how far apart in time two spikes can influence weight change. With the ramp and box kernels, this limit is naturally occurring. For simplifying things further, we normally select symmetric kernels (i.e., with identical duration of the causal and acausal windows) as not to require different STDP timers for each side of the STDP kernel. The second consideration affects the temporal spike interaction and is, in part, addressed by the finite kernel duration since “older” spikes (i.e., spikes which have already left the learning window) can be discarded.

Lastly, throughout this paper we will represent the STDP window duration as *T*_*stdp*_ and the refractory period duration as *T*_*refr*_. Since we are considering implementations on digital neuromorphic systems, both of these duration values are defined as integer multiples of the system time step, Δ*t*. Additionally, it is worth mentioning that there are basically two alternatives for storing spike times: using a bitmap or using multiple timers. In section A1 we detail how the latter is always at least as efficient as the former and, thus, this will be our method of choice throughout the paper. Nevertheless, the proposed STDP learning method using multiple timers can be transferred seamlessly to a bitmap representation of spike times if desired.

### 2.3. Synaptic Weight Data Structures

Storage costs associated to synaptic weight memory arrangements have been previously studied (Moradi et al., 2013; Joshi et al., 2017; Kornijcuk et al., 2018), and here we give an overview of four typical data structures used for representing synaptic weights. We analyze the storage costs (in number of bits) based on number of neurons, weight bit-length, and varying degrees of network connectivity density. Depending on the network topology being emulated, particularly with regards to the connectivity density between pre- and post-synaptic neurons, some of the data structures have clear advantages over the more traditional dense matrix representation. The data structures present common memory tables, which include: adjacency table, pointer table, and weight table. Which tables are used and how they are organized defines the synaptic weight memory arrangement of the network.

As will be presented next, crossbars consume memory even for nonexistent synaptic connections, while pointer-based models store only the existent connections, making them ideal candidates when representing sparsely connected networks. For our analyses, the network connectivity density, *ρ*, represents the percentage of post-synaptic neurons which are connected to a given pre-synaptic neuron, while sparsity can be computed simply as (1−*ρ*). Both crossbars and pointer-based architectures present a weight table (WT) for storing the values of the synaptic weights; however, the latter must (directly or indirectly) also include in WT the address of the post-synaptic neuron associated with each weight, along with an additional memory called the pointer table (PT).

#### 2.3.1. Fully Connected: Crossbar

The most intuitive representation of synaptic weight memory arrangement is by means of a dense matrix, representing full connectivity between the inputs (pre-synaptic neurons) and outputs (post-synaptic neurons). Alternatively, in neuromorphic systems, the dense matrix is sometimes referred to as a *crossbar* (Merolla et al., 2014). In a crossbar, every connection between a pre- and post-synaptic neuron has a reserved space in WT, even if the connection between the neurons does not exist.

An important aspect of WT to consider is that, when using a dense matrix to represent a sparsely connected network, the zero-valued weights can represent either (1) a nonexistent connection or (2) an existent connection with weight currently equal to zero (“inactive”). When simply testing the network (i.e., while not performing synaptic plasticity), both of these cases produce the same results. However, when actually training the network, there should be a distinction between a nonexistent connection and a weight which can momentarily take on the value of zero. To distinguish between these two cases, the first option is to use an additional memory called the adjacency table (AT), where each position *a*_*ij*_ in AT stores a binary value representing the existence (*a*_*ij*_ = 1) or nonexistence (*a*_*ij*_ = 0) of the synaptic connection between pre-synaptic neuron *A*_*j*_ and post-synaptic neuron *B*_*i*_ (Joshi et al., 2017). The second option is to use one of the 2^*W*^ weight values—where *W* represents the bit-length of each weight—to represent a nonexistent connection. The advantage of using this second option is that it removes the memory overhead required for storing AT, thus only using one weight value—instead of an additional bit per weight—to differentiate between existent and nonexistent connections. Throughout our work, crossbars will be implemented using this second option.

**Figure 2:**
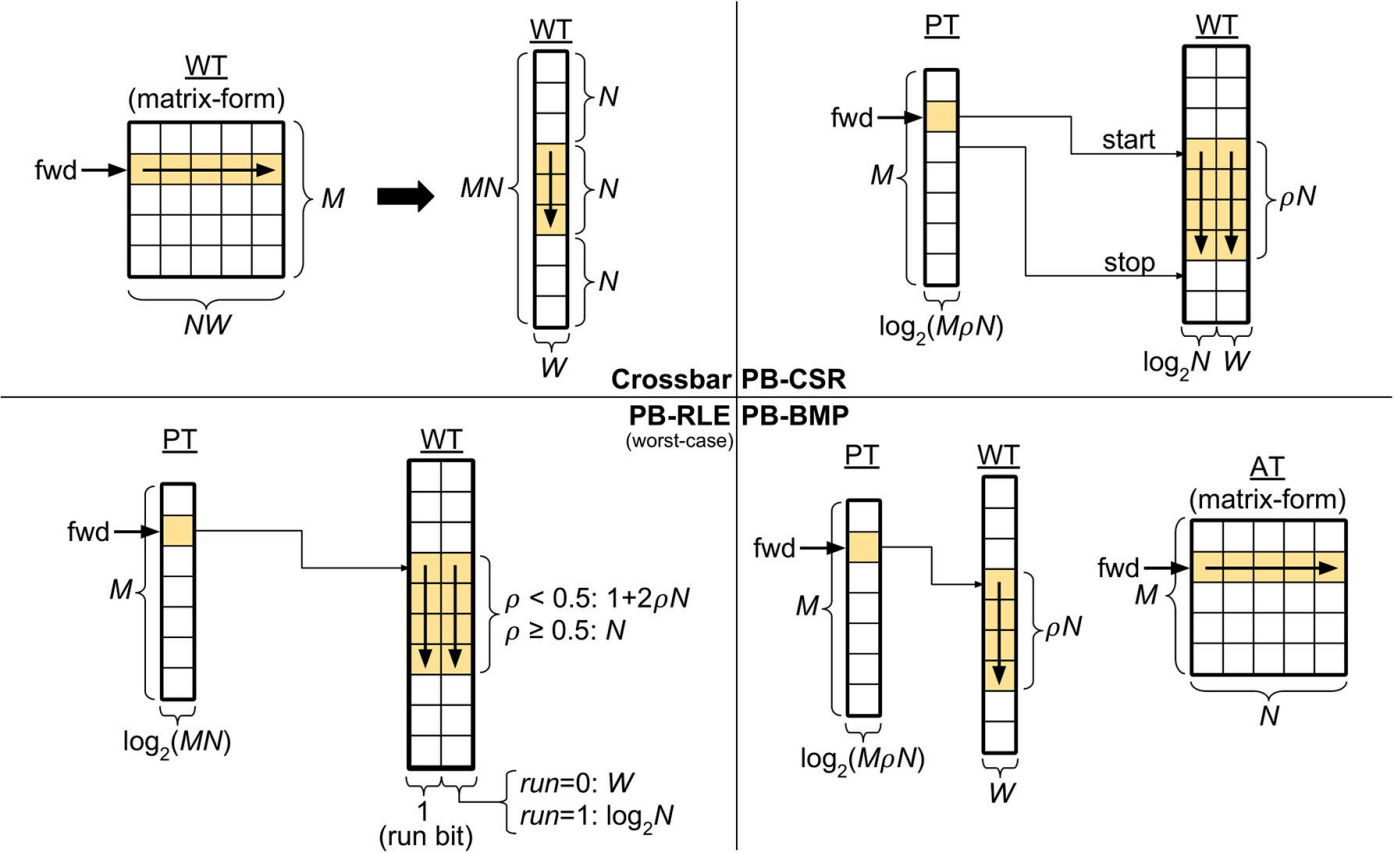
Synaptic weight memory arrangements and storage costs (in bits). Tables: adjacency table (AT), pointer table (PT), and weight table (WT). Parameters: number of pre-synaptic neurons (*M*), number of post-synaptic neurons (*N*), weight bits (*W*), and connectivity density (*ρ*). The forward memory access path has been highlighted. The pointer-based data structures compress data storage, resulting in non-structured solutions which depend on the connectivity and weight distribution in the network. The equations for PB-RLE refer to worst-case scenarios of perfectly interleaved runs and weights.

The top left panel in Figure 2 depicts a crossbar with *M* pre-synaptic and *N* post-synaptic neurons. Though WT can be represented in matrix-form, in the actual memory the weights are stored sequentially, starting with all the weights of pre-synaptic neuron *A*_1_ (i.e., *w*_11_ to *w*_*N*1_), then all the weights of *A*_2_ (i.e., *w*_12_ to *w*_*N*2_), and so forth, until weights *w*_1*M*_ to *w*_*NM*_. Since the crossbar presents a structured WT, the start and stop locations of the weights in WT for each pre-synaptic neuron can be obtained simply by the pre-synaptic address, thus eliminating the need for pointers: the location of the first weight for pre-synaptic neuron *A*_*j*_ can be computed by $A_{j}^{*}=\left(j-1\right)N+1$, with *j* ∈ [1, *M*]. Therefore, forward access in crossbars is performed by starting at address $WT\left(A_{j}^{*}\right)$ and reading *N* consecutive weights. The figure also illustrates forward access (in yellow) for a single pre-synaptic neuron.

#### 2.3.2. Pointer-Based Compressed Sparse Row (PB-CSR)

Using the compressed sparse row (CSR) format (Saad, 2003), each position of WT stores an address-weight pair, (*B*_*i*_, *w*_*ij*_), of the post-synaptic neuron *B*_*i*_ and the respective incoming weight from pre-synaptic neuron *A*_*j*_. In this manner, WT is only populated by existent synaptic connections, and is the most efficient method for storing very sparse networks. The top right panel in Figure 2 exemplifies the PB-CSR model. As shown in the figure, an important aspect of this model is that, when accessing the weights for pre-synaptic neuron *A*_*j*_, since we do not have explicit information of the number of existent connections for this neuron, we must always read the start, *PT*(*j*), and stop, *PT*(*j* + 1), addresses. Therefore, for performing forward access of pre-synaptic neuron *A*_*j*_, start at position $PT\left(j\right)=A_{j}^{*}$ in WT and consecutively read addresses and weights until position $A_{j+1}^{*}-1$. The figure also illustrates the forward path (in yellow) for a single pre-synaptic neuron in PB-CSR, requiring two reads in PT (for start and stop) and *ρN* reads in WT for the existent connections.

#### 2.3.3. Pointer-Based Run-Length Encoding (PB-RLE)

Run-length encoding (RLE) is a method of lossless data compression particularly useful when consecutive sequences of the same value are present (Oliver, 1952). This concept can be used to replace explicit storage of post-synaptic neuron addresses of adjacent nonexistent connections. In PB-RLE, sequences of consecutive nonexistent connections are stored as run counts, and each position in WT stores a “run bit” followed by the run/weight value. A run bit equal to “0” indicates the existence of the synaptic connection, and the value that follows the bit specifies the respective synaptic weight. If the run bit equals “1,” then the data that follows it specifies the run length, representing the number of consecutive post-synaptic neurons which do not have connections with the respective pre-synaptic neuron and are, thus, “skipped” when sequentially reading through WT.

The bottom left panel in Figure 2 illustrates the PB-RLE model. Since the resulting WT after compression depends on the specific distribution of the existent connections in the network, we included equations for the worst-case scenario of perfectly interleaved runs and weights. In other words, for *ρ* < 0.5, no two consecutive positions in WT contain existent connections; for *ρ* ≥ 0.5, no two consecutive connections are nonexistent, resulting in only runs of unit length. The figure also illustrates the forward path (in yellow) for a single pre-synaptic neuron, *A*_*j*_, which consists on starting at position $PT\left(j\right)=A_{j}^{*}$ in WT and consecutively reading weights and processing runs until post-synaptic neuron *N*. When reading the last weight or run, the pointer should be in position $A_{j+1}^{*}-1$ in WT. Forward access requires one read in PT and a variable number of reads in WT, which depends on the distribution of connections between the pre- and post-synaptic neurons. The equations in the figure are defined for the worst-case scenario of perfectly interleaved runs and weights.

#### 2.3.4. Pointer-Based Bitmap (PB-BMP)

Mixing properties of the crossbar and the previous pointer-based data structures, the PB-BMP includes PT, WT, and an additional fully connected adjacency table. As with PB-RLE, bitmaps do not require explicit storage of post-synaptic neuron addresses in WT, while its equivalent run-length encoding is realized via AT. The bottom right panel in Figure 2 illustrates the PB-BMP model and the forward access path (in yellow) for a single pre-synaptic neuron. The start address is stored in PT, and AT stores binary information about connection existence. For forward access of pre-synaptic neuron *A*_*j*_, start the pointer in WT at position $PT\left(j\right)=A_{j}^{*}$, and in matrix-form AT continuously read the entire row *j* in the following manner: for every position in AT which *a*_*ij*_ = 1, read the current weight in WT and move the pointer in WT to the next position; if *a*_*ij*_ = 0, do not change the pointer in WT. After reading the entire row *j* in AT, the pointer in WT should be at position $A_{j+1}^{*}$. The entire forward access requires one read in PT, *N* reads in AT, and *ρN* reads in WT.

#### 2.3.5. Data Structure Storage Costs

When considering a complete neuromorphic system, memory elements must also be accounted for storing neuron variables (e.g., synaptic current, membrane potential, etc.) and the aforementioned STDP timers. However, for a network with *k* pre-synaptic and *k* post-synaptic neurons, the space complexity of storing the synaptic weights is $O\left(k^{2}\right)$, while neuron variables and timers are unique to each neuron and do not depend on the synaptic weight memory arrangement being used, resulting in O(k) space complexity. Therefore, our analyses of memory storage cost and efficiency only incorporate the memory required for storing pointer, adjacency and weight tables, and do not account for the neuron variables and STDP timers.

A summary of the storage costs (in number of bits) for the different synaptic weight memory arrangements is presented in Table 1. The crossbar does not require AT since one of the 2^*W*^ weight values can be used to indicate nonexistent connections. The upper limit of PB-RLE costs vary depending on connectivity density: for *ρ* < 0.5 we considered no two consecutive existent connections, while for *ρ* ≥ 0.5 we considered every run is of unit length. Actual costs for PB-RLE (presented in **Figure 6**) were obtained via simulation, where networks were generated by randomly creating connections based on the value of *ρ*, then producing the respective PT and WT and computing their costs in terms of number of bits required for storage.

**Table 1:** Storage costs (in bits) for different synaptic weight memory arrangements.

**Architecture**		**AT**	**PT**	**WT**
Crossbar^a^		0	0	*MNW*
PB-CSR		0	*M*log_2_(*MρN*)	*MρN*(log_2_*N*+*W*)
PB-RLE^b^	*ρ* < 0.5	0	*M*log_2_(*MN*)	*MρN*(2+log_2_*N*+*W*)+*M*log_2_*N*
	*ρ* ≥ 0.5		*M*log_2_(*MN*)	*MN*(1+(1−*ρ*)log_2_*N*+ρ*W*)
PB-BMP		*MN*	*M*log_2_(*MρN*)	*MρNW*

a The crossbar does not require AT since one of the 2^W^ weight values will be used to indicate nonexistent connections.

b *This is the upper limit of the cost, considering perfectly interleaved runs and weights. More realistic values were obtained via simulation*.

#### 2.3.6. Data Structure Access Costs

Both forward and reverse access to synaptic connections are required for implementing the original STDP learning rule. When a pre-synaptic neuron spikes, we perform forward access in the connectivity table and apply the acausal updates, since this specific pre-synaptic spike must have occurred *after* any post-synaptic spikes which have already taken place. When a post-synaptic neuron spikes, we perform reverse access in the connectivity table and apply the causal updates, since any pre-synaptic spike must have occurred *before* this specific post-synaptic spike.

In the diagrams in Figure 2, the forward (“fwd”) path for accessing weights from pre- to post-synaptic neurons in the weight tables was highlighted in yellow. The structured memory arrangement in crossbars facilitates reverse access by simply performing forward access in the transposed WT. Due to the manner in which weights are stored in memory, pointer-based data structures natively present access only to forward connectivity. For accessing post-to-pre connections (i.e., reverse access), two alternatives are possible: (1) using forward access and sweeping through the entire AT or WT to verify if each pre-synaptic neuron is connected to the post-synaptic neuron of interest or (2) including PT and WT for the reverse connections as well. The first solution does not affect hardware costs, but can be extremely inefficient in terms of computation time (particularly for densely connected networks). The second solution facilitates reverse access by creating explicit tables for this purpose, yet at the cost of basically doubling the memory requirements. In this subsection we will only treat the first option since the second option can be trivially implemented by simply executing forward access on the reverse tables. A final alternative will be presented in section 2.4, where we describe how STDP learning can actually be executed without the need for reverse access, availing of the benefits of pointer-based models (i.e., memory compression and efficient forward access).

An important practical aspect to consider is that memory access in digital memory elements, such as double data rate synchronous dynamic random-access memory (DDR SDRAM), typically occurs in blocks of multiple bytes per read command. Additionally, there is a variable amount of row and column address strobe overhead that precedes the single memory access depending on whether the read is from the same row or from the next column item. For single item accesses, this can add many clock cycles of overhead for reading. Memory controllers can try to optimize memory command scheduling to overcome some of this, but never all of it. Nonetheless, for simplification purposes, in our work we have considered that accessing any single position in memory (to read the value of a single variable) consumes one “computational unit,” and that only one position in memory can be accessed at a time. With this, the computational (or access) cost of performing STDP can be summarized simply by the number of positions in memory which must be accessed to obtain address and weight information for executing the learning rule.

A summary of the access costs for the different synaptic weight data structures is presented in Table 2. In the table, forward costs refer to the average number of positions in the data that must be accessed for a single pre-synaptic neuron, while reverse costs refers to the average number of positions in the data that must be accessed for a single post-synaptic neuron. The equations in the table consider worst-case scenarios for PB-RLE in forward access, as well as worst-case scenarios for all pointer-based data structures in reverse access. Exact closed-form solutions, particularly for reverse access, are difficult to obtain for pointer-based models since the location and distribution of existent connections can greatly impact the data compression, consequently affecting the search for addresses and weights. In any case, since our proposed method removes reverse access altogether, we will focus uniquely on forward access throughout the paper, with the equations in the table merely serving as an assessment of the complexity of reverse access.

**Table 2:** Access costs (per neuron) for different synaptic weight memory arrangements.

**Direction**	**Architecture**		**AT**	**PT**	**WT**
Forward	Crossbar		0	0	*N*
	PB-CSR		0	2	*ρN*
	PB-RLE^b^	*ρ* < 0.5	0		1+2*ρN*
		*ρ* ≥ 0.5		1	*N*
	PB-BMP		*N*	1	*ρN*
Reverse^a^	Crossbar		0	0	*M*
	PB-CSR		0	*M*	*M*(*ρN*)
	PB-RLE	*ρ* < 0.5	0		*M*(1+2ρ*N*)
		*ρ* ≥ 0.5		*M*	*MN*
	PB-BMP		*M*+*ρM*(*N*−1)	*ρM*	*ρM*

a The equations for the pointer-based models consider worst-case scenarios. The values presented in Figure 6 were obtained via simulation.

b *This is the upper limit of the cost, considering perfectly interleaved runs and weights. More realistic values were obtained via simulation*.

### 2.4. STDP Learning Rule With Forward-Only Connectivity Access

Based on the equations presented in Table 2, reverse access in pointer-based data structures can be quite inefficient. Because of this limitation, multiple efforts have been made in approximating STDP learning using forward-only connectivity, including simplifying the STDP rule by equally updating all the synaptic weights based on recent spike activity, using other variables as a proxy for the post-synaptic spike times when computing causal updates, and delaying the weight updates. Our method falls under the latter category; however, contrary to these approximate alternatives, it can produce exact equivalence to STDP, as will be shown in the Results section.

When using pointer-based data structures for storing synaptic weights, acausal updates can be immediately performed at the onset of a pre-synaptic spike using forward connectivity access of PT. Causal STDP updates, however, should be performed at the onset of post-synaptic spikes, requiring reverse connectivity access. Since pointer-based models natively have only forward connectivity access, we have devised a method which performs causal updates at the onset of yet another pre-synaptic neuron event: the STDP timer expiration. Therefore, instead of immediately applying the causal updates at the onset of post-synaptic spikes, the update is delayed until the pre-synaptic STDP timer expires, at which point the causal influence of a spike ceases. The two types of weight updates in our proposed algorithm are described below:

- **Acausal update:** At the onset of a pre-synaptic spike from neuron *A*_*j*_, perform forward access in WT starting at position $PT\left(j\right)=A_{j}^{*}$, and verify the STDP timers of the post-synaptic neurons connected to *A*_*j*_. For every post-synaptic neuron which has spiked not long ago (i.e., with an active STPD timer), perform the acausal weight update.
- **Causal update:** At the moment of expiration of the pre-synaptic STDP timer of neuron *A*_*j*_, perform another forward access in WT starting at position $PT\left(j\right)=A_{j}^{*}$, once again verifying the STDP timers of the post-synaptic neurons connected to *A*_*j*_. For every post-synaptic neuron which has recently spiked (i.e., with an active STPD timer), perform the causal weight update.

**Figure 3:**
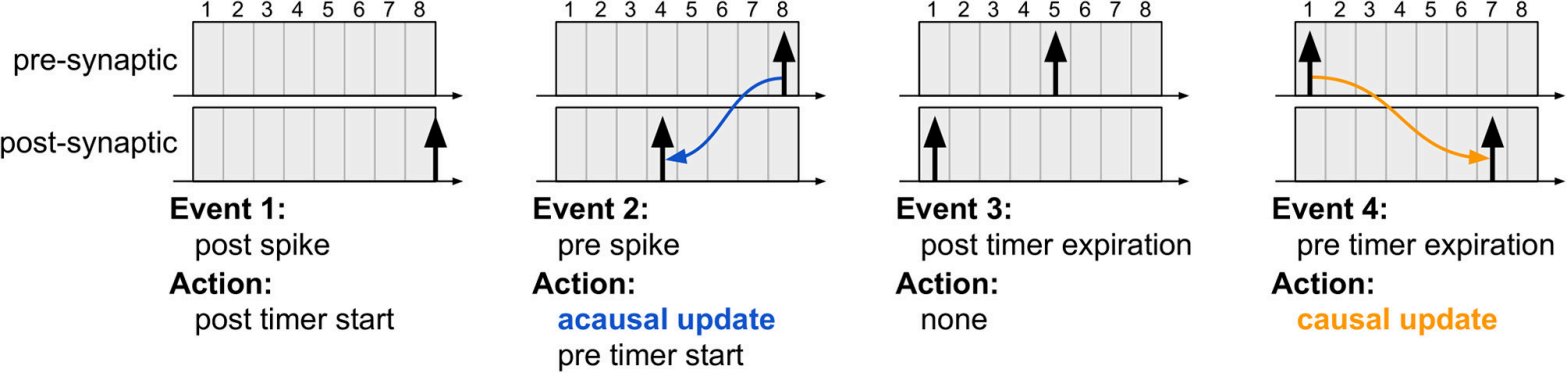
The four typical events which occur during the proposed STDP learning algorithm. The first event illustrates post-synaptic spike generation, while the third event is the moment a post-synaptic spike exists the learning window (i.e., its STDP timer expires); in both cases, no weight updates are performed since the algorithm is driven only by pre-synaptic events. The second event illustrates pre-synaptic spike generation, resulting in acausal update. The fourth event illustrates pre-synaptic STDP timer expiration, resulting in causal update. Note that the post-synaptic spikes in the third and fourth instants are distinct spike events, used to highlight that acausal and causal updates can take place between the same pair of neurons depending on the order of the spikes.

For clarifying the proposed algorithm, Figure 3 illustrates four different instants during system evolution for a causal and an acausal STDP window duration of 8 time steps each. These events are described below:

1. The first event illustrates a new post-synaptic spike, at which time this neuron's STDP timer is initialized and no weight updates occur.
2. In the second event, the pre-synaptic neuron elicits a new spike, initializing its STDP timer and also performing the acausal weight update. This update is performed just as it would be in the original STDP algorithm via forward connectivity access.
3. The third event illustrates the expiration of the post-synaptic STDP timer. No action is required since the acausal update of its weight has already been serviced.
4. In the fourth event, the pre-synaptic STDP timer expires, at which point the causal weight update takes place. Unlike the original STDP algorithm, in which causal updates would have taken place at the onset of a post-synaptic spike, the proposed method delays the update until the pre-synaptic STDP timer expires, requiring, therefore, only a second forward access and avoiding reverse connectivity access altogether.

Using our method, if every neuron is configured to be able to spike at most once during the STDP window, then the weight updates will always fall under one of these four scenarios and produce results which exactly match those obtained by the original STDP algorithm (this will be shown in section 3.3). However, if a neuron is allowed to spike multiple times during *T*_*stdp*_, then many different scenarios may arise between the moment a post-synaptic neuron spikes and the moment the STDP timer of its pre-synaptic neuron expires. In this case, the proposed method may incur in incorrect weight updates, as shown next.

#### 2.4.1. Drawbacks of Allowing Multiple Spikes Inside the STDP Window

If the system is designed without guaranteeing that no neuron spikes more than once inside its STDP window, some natural drawbacks arise. Below we list these cases to better illustrate the importance of the two criteria— three of the drawbacks present direct solutions, while the fourth does not. To generate these specific cases, we will consider nearest-neighbor temporal spike interaction (where only the nearest spikes are considered; refer to subsection 2.4.3), and we will configure the neurons with *T*_*refr*_ < *T*_*stdp*_ and use a single timer of length ⌈log_2_(*T*_*stdp*_ + 1)⌉ bits per neuron.

**Figure 4:**
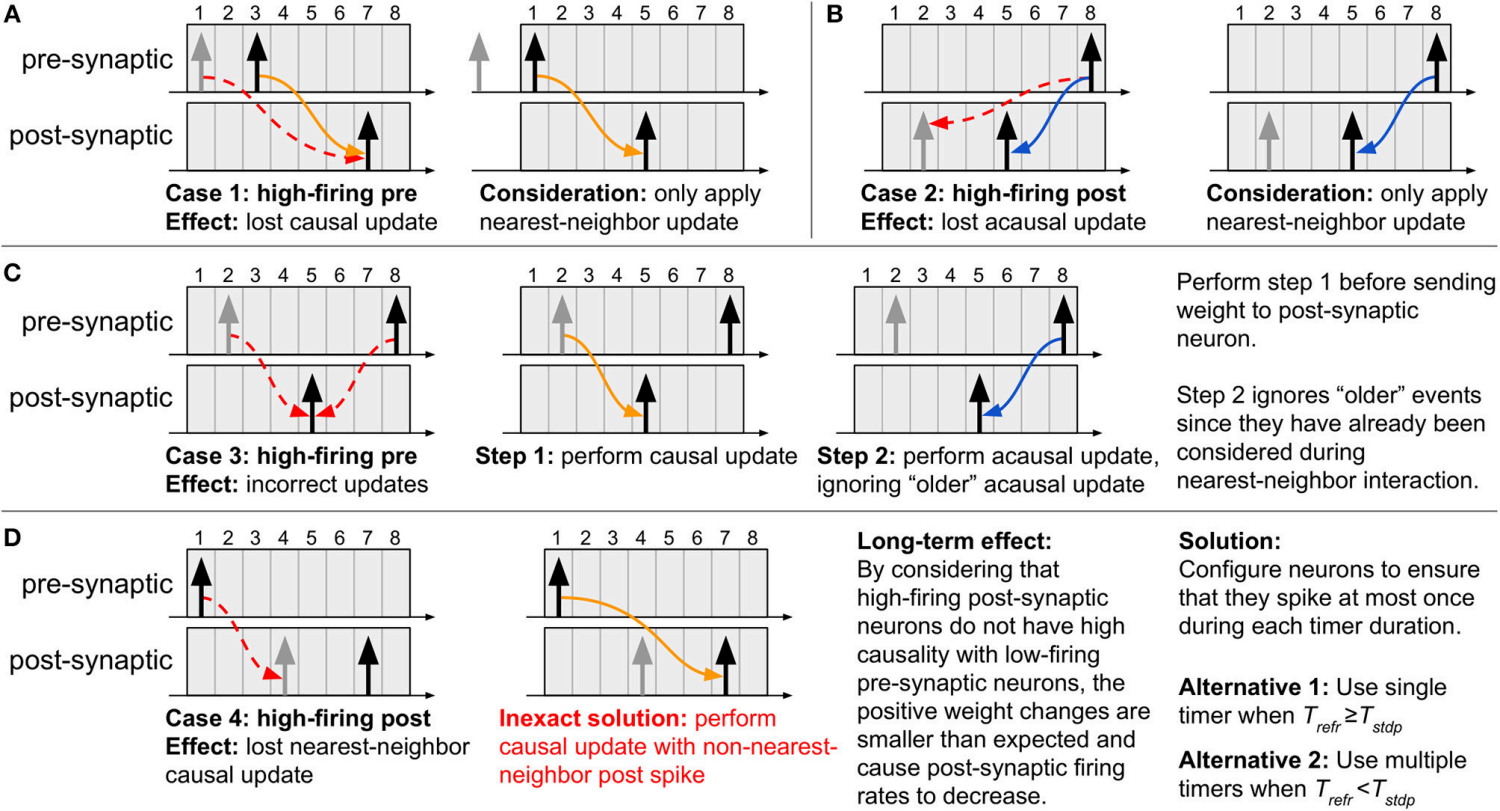
Special cases which arise when using a single timer and *T*_*refr*_ < *T*_*stdp*_. **(A–C)** By considering nearest-neighbor temporal spike interaction, cases 1–3 can be correctly addressed, **(D)** yet case 4 does not present a direct solution. To overcome all the drawbacks inherent to the proposed method, the neurons in the system must be configured as to ensure that they spike at most once during each timer duration. If the neurons can be configured with *T*_*refr*_ ≥ *T*_*stdp*_, then we guarantee that only one spike can occur inside the STDP window. However, if the neurons present *T*_*refr*_ < *T*_*stdp*_, then the only manner of capturing all the spikes is to use multiple timers for the STDP window, each with duration *T*_*refr*_.

**Case 1: High-firing pre-synaptic neuron (refer to Figure 4A):** If a second pre-synaptic spike occurs while the first spike is still inside the STDP window, the timer will be restarted and information about the first spike will be lost. Since the post-synaptic spikes occur after the second pre-synaptic spike, the correct update will take place since only nearest-neighbor influence is considered.

**Case 2: High-firing post-synaptic neuron (refer to** **Figure 4B****):** If a second post-synaptic spike occurs before the pre-synaptic spike, information about its first spike time will be lost. Since the pre-synaptic spike occurs after the second post-synaptic spike, once again the correct update will take place since only nearest-neighbor influence is considered.

**Case 3: High-firing pre-synaptic neuron (refer to** **Figure 4C****):** If a second spike occurs for a pre-synaptic neuron whose STDP timer has not yet expired, then the timer will be restarted and information about the first spike will be lost. As a solution, first service the pending causal updates (relative to the first spike), then service the acausal updates (relative to the second spike) only for post-synaptic spikes which have occurred after the first pre-synaptic spike. The reason for this is that the acausal updates of post-synaptic spikes older than the first pre-synaptic spike have already been performed at the onset of this first spike. Lastly, restart the STDP timer for the new spike.

**Case 4: High-firing post-synaptic neuron (refer to** **Figure 4D****):** If we have a post-synaptic neuron which spikes frequently (i.e., before the pre-synaptic timer expires and the causal updates are performed), then the nearest-neighbor spike information between pre- and post-synaptic neurons will be lost and overwritten by the new post-synaptic spike time (since the post-synaptic STDP timer is restarted). An objective, yet inexact, solution is to simply ignore this issue given that a single pre-synaptic spike should not have a strong causal relation with a high-firing post-synaptic neuron. With this, a causal update will still take place at the expiration of the pre-synaptic STDP timer, except it will just not be with the nearest-neighbor post-synaptic spike. To prevent this scenario from occurring, we must ensure that a maximum of a single spike can occur in the duration of each timer, demanding that the system be designed as presented next.

#### 2.4.2. Criteria for Exactness Between Methods

The effect of not being able to implement nearest-neighbor causal updates has the effect of the weights not increasing as much as expected, resulting in lower synaptic efficacy and, consequently, fewer post-synaptic spikes. For the results of the proposed method to exactly match those obtained by the original STDP algorithm, each neuron must present one timer per refractory period, capturing every possible spike, and resulting possibly in multiple timers to cover the entire duration of the STDP learning window. In other words, we must use ⌈*T*_*stdp*_ / *T*_*refr*_⌉ timers, each of length ⌈log_2_(*T*_*refr*_ + 1)⌉ bits. Note that if *T*_*refr*_ ≥ *T*_*stdp*_, this reduces to the expected single timer of length ⌈log_2_(*T*_*refr*_ + 1)⌉ bits. This rule has the advantage of allowing different types of temporal spike interaction (see subsection 2.4.3).

Details of the multi-timer method are presented in Appendix A1 and shown in Figure A1. To implement our proposed method of STDP learning using multiple timers, we must simply treat each individual timer as was done in Figure 3. The causal updates, however, can be implemented in two different manners.

1. During the traversal of the spike through the timers, at the instant of timer expiration the causal updates are performed between the current spike and all “newer” post-synaptic spikes. This means that whenever any of the multiple pre-synaptic timers expires, perform weight updates with the post-synaptic spikes which have recently entered the queue—meaning we must verify only the first timer of the post-synaptic neurons.
2. The second option implies in performing the causal update only when the *T*-th (i.e., the last) pre-synaptic timer expires. This method has the advantage of possibly incurring only two instants of updates: when the spike enters and when it exists the spike history queue. However, if a new pre-synaptic spike occurs while a spike is still traversing the queue, then the causal weight updates between the first spike and any post-synaptic spikes that occurred after it must be performed prior to updating the post-synaptic neuron variables. This effect is similar to that of case 3 in Figure 4.

It may appear at first glance that both of these alternatives incur in more memory access than the original STDP algorithm. The first method can, in fact, produce more updates than the second alternative, particularly for sparse pre-synaptic activity—though it is a more systematic way of implementing updates since we must only verify the first timers for the post-synaptic neurons. The second alternative, however, implements updates only when actually required, consuming (on average) the same number of memory accesses as the original STDP learning rule. This can be elucidated by considering the case of a high-firing post-synaptic neuron: the original algorithm would search through all its pre-synaptic neurons even if most have not spiked, while the proposed algorithm would only verify the pre-synaptic neurons which have recently spiked and could, therefore, have some causal influence on the post-synaptic spikes. If we consider the case of a high-firing pre-synaptic neuron, then the inverse is valid, thus resulting most likely in a similar average cost for both methods.

#### 2.4.3. Temporal Spike Interaction

**Figure 5:**
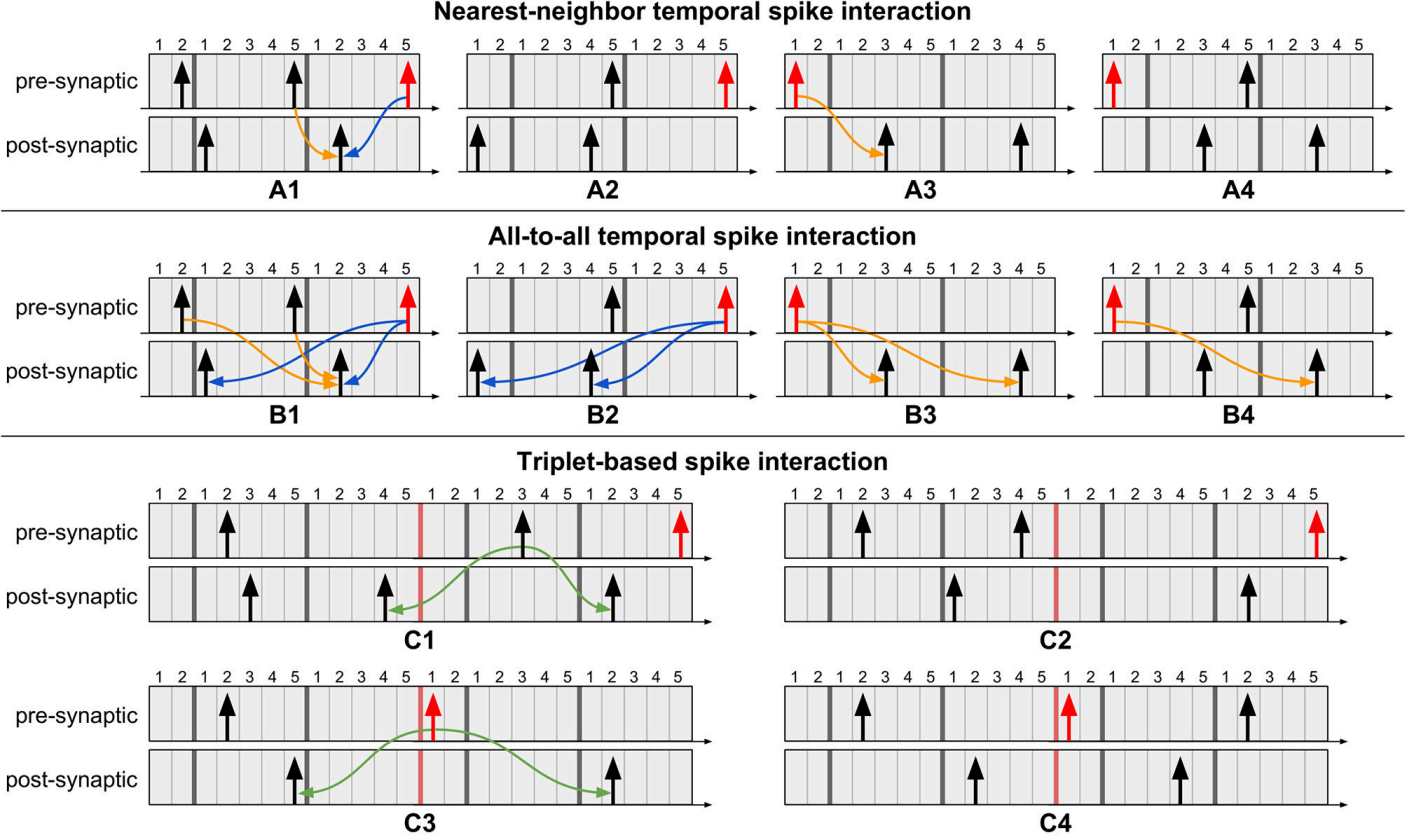
Example scenarios of proposed method for different types of temporal spike interactions using multiple timers. Pre-synaptic spikes in red represent event of interest: a new spike event or the last STDP timer expiration event. **(A1)** Perform pending causal update before acausal update. **(A2)** No updates required since no post-synaptic spikes occurred after the previous pre-synaptic spike. **(A3)** Perform causal update. **(A4)** No update required because the causal update was performed when the most recent pre-synaptic spike occurred. **(B1)** Perform causal updates with post-synaptic spikes which occurred after the previous pre-synaptic spike, followed by the acausal updates. **(B2)** No causal updates required since no post-synaptic spikes occurred after the previous pre-synaptic spike. **(B3)** Perform causal updates. **(B4)** Only a single causal update is required since the other causal update was performed when the most recent pre-synaptic spike occurred. **(C)** Triplet-based spike interaction requires spikes to be stored for a longer duration. For pre-synaptic spikes, the timers to the right of the red bar represent the active region. **(C1)** Perform triplet update since the previous pre-synaptic spike is still in the active region. **(C2)** No triplet update required since the previous pre-synaptic spike is already in the inactive region (i.e., left-side timers). **(C3)** Perform triplet update at expiration of pre-synaptic STDP timer. **(C4)** No triplet update required since it was performed when the most recent pre-synaptic spike occurred.

Temporal spike interaction can go to the extreme of considering only the nearest spikes, known as *nearest-neighbor interaction* (Morrison et al., 2008). At the other extreme, *all-to-all interaction* considers influence of the entire spike history. A third variant is a *triplet-based interaction* (Pfister and Gerstner, 2006), where a sequence of post-pre-post spikes, for example, is a template for updating weights. Examples illustrating these temporal spike interactions using multiple timers for *T*_*stdp*_ = 12 and *T*_*refr*_ = 5 are presented in Figure 5. The procedure when using multiple timers follows that of a single timer: weights are updated at the onset of a new pre-synaptic spike and at the expiration of the (last) pre-synaptic STDP timer. Note in Figure 5C that the triplet-based interaction requires spikes to be stored for a longer duration since the “older” post-synaptic spike in the post-pre-post triplet may already have left its active region (i.e., the timers to the right of the red bar), but is still of use for an active pre-synaptic spike. From the figure we show that, independently of the type of temporal spike interaction being implemented, as long as the appropriate number of timers is used and we address the pending causal updates before sending the weights to the post-synaptic neurons (as per case 3 in Figure 4C), then our method produces exact equivalent results to original STDP.

## 3. Results

### 3.1. Data Structure Efficiency

**Figure 6:**
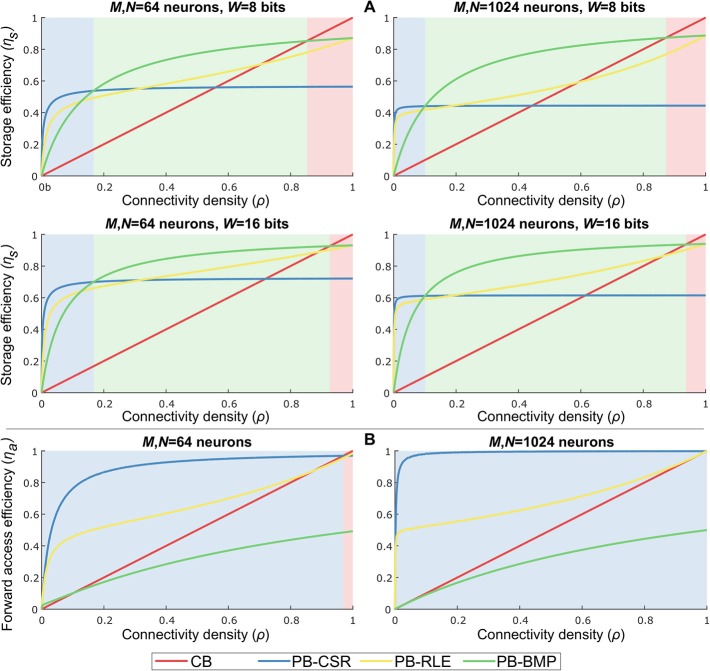
Data structure storage and forward access efficiencies for different parameter settings and varying connectivity density. Parameters: number of pre-synaptic neurons (*M*), number of post-synaptic neurons (*N*), bits per weight (*W*), and connectivity density (*ρ*). The light-shaded regions behind each plot indicate the most efficient model for specific values of *ρ*. **(A)** Storage efficiency in pointer-based models is higher than in crossbars for nearly all values of *ρ*. Increasing weight bit-length is more impactful than increasing network size. PB-BMP efficiency improves for larger networks since it does not explicitly store post-synaptic addresses (except as binary values in AT). All data structures show higher efficiency for larger networks. **(B)** Forward access is efficiently performed in pointer-based models due to their compression mechanism, particularly in sparsely connected networks. PB-CSR has advantage over the other models throughout most values of *ρ* because it does not require data decompression to obtain address-weight pairs.

Based on the data structure storage and access costs, a comparison of storage and forward access efficiencies for multiple network sizes, weight bit-lengths, and connectivity densities is shown in Figure 6. By varying the number of pre-synaptic (*M*) and post-synaptic (*N*) neurons, the connectivity density (*ρ*), and the number of bits used to represent each weight (*W*), we empirically verified the performance of each data structure for different network configurations. For each data structure, storage cost, *C*_*s*_, is compared to the reference cost value, $C_{s}^{\text{ref}}=M\rho NW$, representing the amount of memory required to store the weights of only the existent connections in the network. Storage efficiency is then computed as $\eta_{s}=C_{s}^{\text{ref}}/C_{s}$. Forward access cost, *C*_*a*_, is compared to the reference computational cost value, $C_{a}^{\text{ref}}=\rho MN$, representing the total number of variables to be accessed when reading data for all pre-synaptic neurons once (i.e., obtaining the entire network address-weight pairs). Forward access efficiency is then computed as $\eta_{\text{a}}=C_{a}^{\text{ref}}/C_{\text{a}}$. The results in the plots were obtained by generating 1,000 randomly connected networks according to the parameter set, and averaging the costs of these networks per connectivity density. The light-shaded regions behind each plot indicate the model with the highest efficiency for specific values of *ρ*.

As we can observe in Figure 6A, pointer-based models have a great advantage over crossbars due to their data compression, with the PB-BMP model showing the best overall performance for a large range of *ρ*. Naturally, for larger weights, pointer-based models show a greater advantage, particularly for sparsely connected networks (i.e., small values of *ρ*). Increasing network size has only a slight impact on PB-BMP models, since in these models the only additional memory required beyond the reference value is the rather low-cost AT. Conversely, PB-CSR and PB-RLE are clearly affected when mapping larger networks since they directly (for PB-CSR) or indirectly (in run-lengths for PB-RLE) must store larger post-synaptic addresses in WT. For forward access, Figure 6B shows that pointer-based models PB-CSR and PB-RLE have a natural advantage over the other two models since they do not require reading every position in their tables. Between these two models, PB-CSR performs better than PB-RLE (except for *ρ* = 1) because the latter requires decompressing the data by reading run-lengths, while the former requires only two read commands in PT (the start and stop addresses) along with the *ρMN* weights to be read. The PB-BMP model can achieve a maximum efficiency of about 50% because it requires two read commands per existent connection: one read in AT to identify if the connection exists and one read in WT to find the weight value of the connection. The performance of the crossbar grows linearly with connectivity density, and is efficient at very large values of *ρ*.

### 3.2. Budget Efficiency

In order to identify the optimal solution for a given implementation budget in terms of memory storage and computational effort (i.e., memory accesses), we defined the *budget efficiency* metric as η = *λ*η_*s*_ + (1−*λ*)η_*a*_, where η_*s*_ is storage efficiency, η_*a*_ is forward access efficiency, and *λ* is a tunable parameter defining the storage-versus-access trade-off. Note that η_*a*_ is computed as the forward access efficiency since (1) both causal and acausal updates only require this type of access in pointer-based models and (2) reverse access in crossbars is just as efficient as forward access.

**Figure 7:**
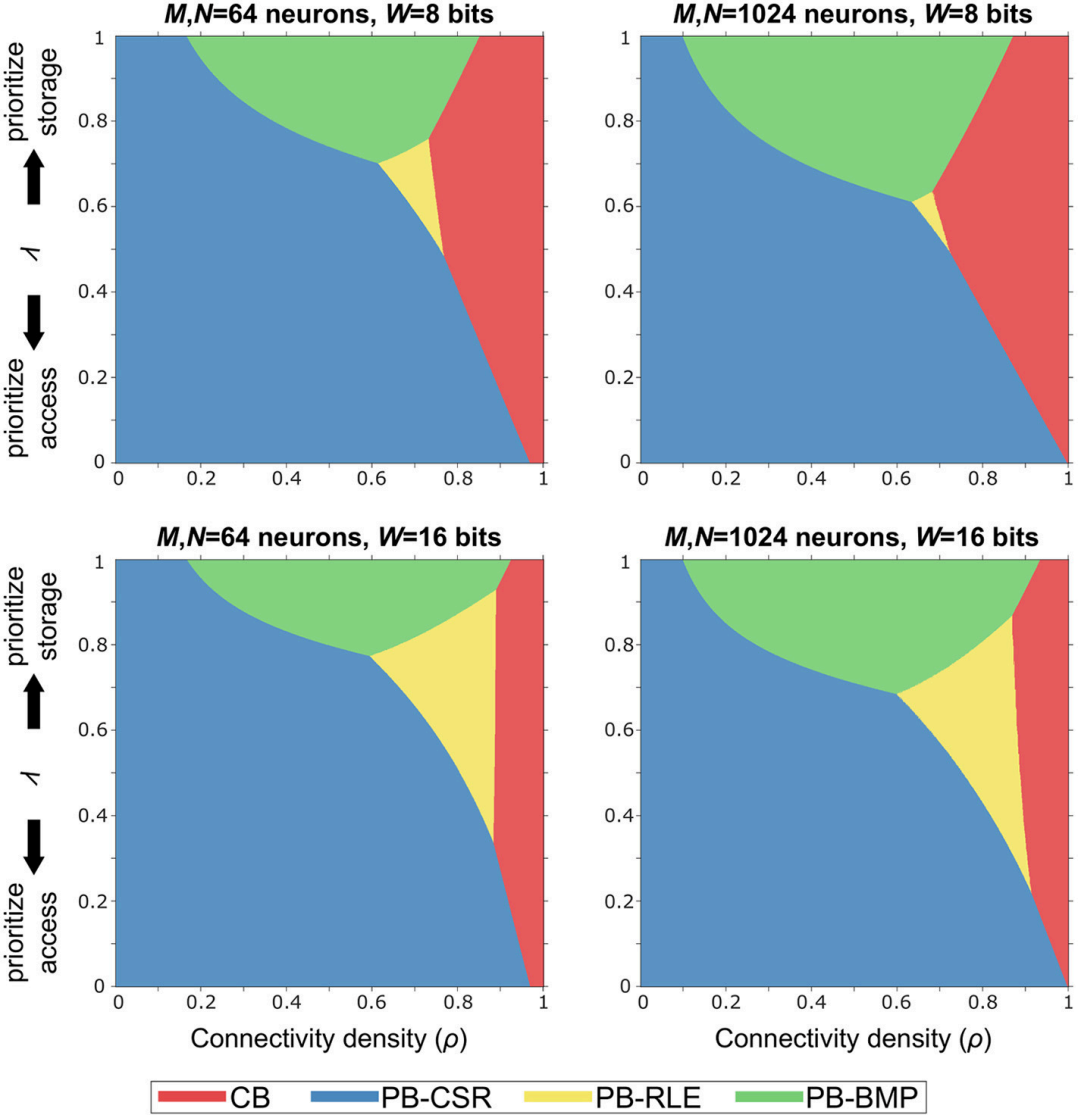
Budget efficiency, η = *λ*η_*s*_ + (1−*λ*)η_*a*_. Parameters: η_*s*_ is the storage efficiency, η_*a*_ is the forward access efficiency, and *λ* is a tunable parameter defining the storage-versus-access trade-off. Given efficiency priority and overall network connectivity density, the optimal memory arrangement for synaptic weights can be obtained. Pointer-based models cover most of the range of values for *ρ* and *λ* because the proposed STDP algorithm takes advantages of their efficient memory compression and forward access.

The graphs in Figure 7 illustrate the optimal models (based on the shaded colors) for different network parameter settings in the *ρλ*-plane. For networks where memory access efficiency is priority (i.e., small values of *λ*) and/or for sparse networks (i.e., small values of *ρ*), the PB-CSR model is the clear optimal solution. This is mainly due to the compression method in PB-CSR, where no AT and no decompression (as in PB-RLE) are required, making weight storage simple and forward access efficient. However, when memory storage is priority (i.e., for large values of *λ*), the PB-BMP model spans the longest range of connectivity densities as the optimal solution. For densely connected models, the crossbar appears as the best alternative since the nonexistent connections entail only a small amount of storage overhead, while presenting efficient forward access. Interestingly, the PB-RLE model spans only a small region close to the center of the graph (especially for small weight bit-lengths), resulting as the optimal solution for more specific cases of *ρ* and *λ*.

### 3.3. Proof-of-Concept Example

Many of the examples and results presented thus far throughout our work were obtained via simulation of various network topologies and connectivity distributions. In this section, we present an additional example to highlight the equivalence of our proposed algorithm with the original STDP learning rule—when implementing one of the two criteria presented in subsection 2.4.2. The effect of case 4 from subsection 2.4.1— where nearest-neighbor causal updates are lost—will be demonstrated, along with an example of all-to-all temporal spike interaction which perfectly matches the original STDP algorithm.

The experimental setup involves 256 post-synaptic neurons receiving spike inputs from 256 pre-synaptic neurons. Initial weight values were sampled from a Gaussian distribution with 0.1 mean and unit variance. All the neurons were configured with symmetric STDP ramp kernel of window duration of *T*_*stdp*_ = 16 and maximum weight change of ±0.01, spiking threshold of *V*_*th*_ = 1.0, and refractory period duration of *T*_*refr*_ = 4. Pre-synaptic neurons were set with spiking probability of 10% when outside the refractory period. The leaky integrate-and-fire neuron model was used for the post-synaptic neurons, governed by the equation $V_{i}\left(t+1\right)=\alpha V_{i}\left(t\right)+\underset{j}{\sum }w_{ij}s_{j}\left(t\right)$, where the membrane memory constant, α was set to 0.9. The network dynamics were simulated for 1, 000 time steps, during which all the weights and membrane potentials were recorded at each time step. Since causal weight updates occur at different instants of the algorithm for the original STDP learning rule and our proposed method, directly observing the weight values at each time step for such a large number of weights is not feasible. Therefore, to validate our method, we compared the post-synaptic membrane potentials for each neuron throughout the entire simulation. Additionally, for completeness, the post-synaptic spiking activity was analyzed by computing the distance between the van Rossum spike traces (Rossum, 2001) for the two algorithms. The time constant of the exponential kernel for generating the continuous traces was set as the time constant of the membrane potential and computed as τ_*R*_ = −1/log(α) ≈ 9.5.

**Figure 8:**
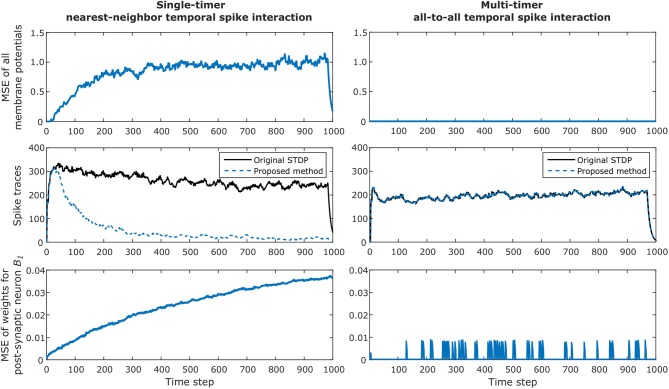
Example of convergence of proposed method for STDP learning for a network with 256 pre-synaptic and 256 post-synaptic neurons, configured with *T*_*stdp*_ = 16 and *T*_*refr*_ = 4. The left column illustrates results when one timer is used and simply nearest-neighbor interaction is considered. The right column illustrates results when multiple timers (in this case, 4 timers) are used to capture all possible spikes which can occur inside the STDP window. The top row shows how the total mean squared error (MSE) of all post-synaptic membrane potentials between the original STDP algorithm and our method diverges when using only one timer; this is caused by the effect described in case 4 in section 2.4.1. For the multi-timer solution, the membrane potentials always match, and the resulting MSE is zero. The second row shows the continuous van Rossum spike traces, where the effect of smaller weight updates in the case of using a single timer is clearly observed by the decreasing post-synaptic spike activity over time. As expected, the multi-timer solution produces post-synaptic spikes identical to those obtained by the original STDP algorithm. The bottom row illustrates the MSE of all incoming weights for post-synaptic neuron *B*_1_. Once again the lost causal nearest-neighbor updates make the weights diverge for the single timer solution; for the multi-timer solution, we can see that the MSE temporarily increases but soon after returns to zero, which is simply the effect of the delayed causal updates.

The simulation results for the network are presented in Figure 8, where we verify the convergence of our proposed method for STDP learning. The left column illustrates results when one timer is used and simply nearest-neighbor interaction is considered for the original algorithm and our method. The right column illustrates results when multiple timers (in this case, 4 timers) are used to capture all possible spikes which can occur inside the STDP window and all-to-all spike interaction is performed for the original algorithm and our method. Note that the single-timer and multi-timer results were obtained from different simulations since only one temporal spike interaction can be considered at a time.

The top row shows how the total mean squared error (MSE) of all post-synaptic membrane potentials between the original STDP algorithm and our method diverge when using only one timer; this is the effect described in case 4 in subsection 2.4.1, where post-synaptic weights receive smaller causal updates than expected. For the multi-timer solution, the membrane potentials always match those obtained by the original STDP algorithm, and the resulting MSE is zero.

The second row shows the total van Rossum spike traces obtained by adding all traces after passing each spike through the exponential kernel. In this example, the effect of smaller weight updates because of lost causal nearest-neighbor updates is clearly observed by the decreasing post-synaptic spike activity when using a single timer. As expected, the multi-timer solution produces post-synaptic spikes identical to those obtained by the original STDP algorithm.

Lastly, the bottom row illustrates the MSE of all incoming weights for post-synaptic neuron *B*_1_. Once again, the effect of case 4 causes the weights to diverge for the single-timer solution. For the multi-timer solution, we can see that the MSE momentarily increases but soon after returns to zero; this effect occurs because of the delayed causal updates, but always produces the correct weight at the moment the weight must be effectively used. Note in the graphs that in the last *T*_*stdp*_ time steps the membrane potentials and spike traces for the single timer method also converge to zero, simply because we enforced all pre-synaptic neurons to stop spiking during this duration for the final weights obtained by the multi-timer solution to exactly match those of the original STDP algorithm at the last simulation time step (i.e., so the delayed causal updates could be completed and all timers could return to zero).

## 4. Discussion

Storage costs associated to synaptic weight memory arrangements have been previously studied. In Moradi et al. (2013), the authors describe a network clustering scheme which uses a two-stage routing architecture to reduce the overall memory storage requirements. This method is also mentioned in Joshi et al. (2017) and is referred to as “clustered addressing.” In both of these studies, the storage savings comes at the cost of reduced flexibility in network connectivity, since a specific topology must exist for groups of neurons to be clustered together. Instead, we decided not to constrain our networks to any structured topology. In Joshi et al. (2017), the authors describe the data structures we have presented, highlighting, particularly, the storage cost savings obtained for a large range of connectivity density when using the PB-BMP architecture. However, the impact of pointer-based models on learning algorithms was only briefly mentioned, and memory access costs were not analyzed. More recently, the impact of using different memory arrangements on spike routing and network traffic congestion was described in Kornijcuk et al. (2018). Though the work describes a theoretical means of routing-rate evaluation and results for maximum network sizes for each of their memory arrangements, it does not target any specific learning algorithm, and the experimental results focus only on an inference task without synaptic plasticity. More recently, the authors in Kim et al. (2018) proposed a modified SRAM which enables transposable memory access. The method is interesting as it facilitates the reverse (post-to-pre) access for causal updates; however, it can only be applied to fully connected network topologies (i.e., crossbars), and, thus, are not efficient for representing sparse networks since compressed data structures are typically not transposable.

In terms of spike-driven learning, there have been multiple attempts to replicate or approximate STDP with forward-only connectivity. The motivation for storing synaptic weights in a pre-synaptic perspective (i.e., pre-to-post) is because post-synaptic-driven systems are not as efficient in terms of number of memory accesses as pre-synaptic-driven systems; this is mainly because, as we sweep through neurons to update their states during a system time step, Δ*t*, for each post-synaptic neuron we must verify the spike state of every pre-synaptic neuron, even if none of these has spiked. Conversely, pre-synaptic-driven systems operate in an on-demand fashion, accessing the pre-synaptic spike states only as needed.

In Pedroni et al. (2016); Detorakis et al. (2018), we described a less-detailed version of our method; yet, we did not study all the data structures nor were we able to address all of the drawbacks incurred by delayed causal updates (as we have shown in the current paper). One of the earliest works which evaluated the complexity of implementing the STDP learning algorithm in a neuron address domain was presented in Vogelstein et al. (2003). The authors discussed how the address-event representation (AER) protocol could support STDP learning in the address domain. Being pioneering work, the paper considered only small networks, consequently not addressing the different possible arrangements for organizing synaptic weights in memory and the implications of requiring reverse access for performing causal updates.

Methods that approximate STDP learning by equally updating all the synaptic weights based on recent spike activity have been proposed. In Bichler et al. (2012), the authors use a special form of STDP which equally depresses all the synapses that did not recently contribute to the post-synaptic spike activation regardless of their activation time; in contrast, synapses that were activated with a pre-synaptic spike a short time before post-synaptic spikes are strongly potentiated. The authors in Yousefzadeh et al. (2017) created a more hardware-friendly version of this model by limiting the number of synapses to be potentiated (instead of limiting the STDP time window duration), eliminating the need for time-stamping the spikes. Though efficient in terms of memory access, with both of these methods it is not possible to depress synapses whose activation time is precisely not correlated with the post-synaptic spike, and the methods only work if LTD is systematically applied to synapses not undergoing an LTP. Additionally, the methods are post-synaptic-driven, undergoing the aforementioned drawbacks of this mechanism.

Another alternative to approximating STDP is by using other variables (usually post-synaptic membrane potential) as a proxy for the post-synaptic spike times when computing causal updates. This learning rule was proposed in Brader et al. (2007) and has even been incorporated in the SpiNNaker system (Davies et al., 2012; Lagorce et al., 2015). More recent work describes how to use the rule for learning sequences of spikes (Sheik et al., 2016). Once again, though very efficient in terms of memory access and spike time storage, in this method exact STDP is not possible as post-synaptic potential serves only as a [deterministic (Lagorce et al., 2015) or probabilistic (Sheik et al., 2016)] proxy of the post-synaptic spike time and, in many cases, is not capable of capturing the subtle spike time causalities of STDP.

The third category of methods for approximating STDP consists on delaying the weight updates, and is the category which our proposed method falls under. In the Loihi system, the authors adopt a less event-driven method where synaptic modification is performed in an epoch-based mechanism (Davies et al., 2018). Their method delays the updating of all synaptic states to the end of a periodic learning epoch time, and, to avoid receiving more than one spike in a given epoch, the epoch period is normally set to the minimum refractory delay of all neurons in the network. Though Loihi implements forward connectivity tables for supporting generalized STDP rules, the periodic servicing (i.e., non-event-driven methodology) can result in inexact weights being delivered to post-synaptic neurons since multiple pre-synaptic spikes may occur before a weight update takes place. Therefore, certain conditions in firing rates must be guaranteed for their method to be equivalent to STDP.

In the current version of the SpiNNaker system, STDP learning is approximated using a trace-based approach via delayed updates (Mikaitis et al., 2018). Since in trace-based STDP each spike leaves an exponentially decaying trace (Morrison et al., 2008), this renders possible linearly accumulating the spike traces into a single variable, representing the total current effect of all past spikes. In this manner, weight updates can then be performed in an online fashion at the onset of either pre- or post-synaptic spikes. In SpiNNaker, however, the updates only occur at the onset of pre-synaptic spikes, meaning that, for the method to follow rather closely to original STDP, the system relies on frequently firing pre-synaptic neurons. This issue can be observed in the case when a post-synaptic neuron spikes multiple times soon after a pre-synaptic spike (typically resulting in large causal updates): if the pre-synaptic neuron spikes again in a much later time, then the causal updates will be practically null due to the almost completely decayed traces (somewhere along the lines of the problem encountered in case 4 in Figure 4D). Additionally, besides serving only as an approximation to STDP, the trace-based method requires an exponentially decaying kernel, and, thus, other kernels such as those in Figure 1C cannot be implemented.

Perhaps the most similar work to ours has been presented in Jin et al. (2010), which uses a deferred-event approach and stores spike times for postponed processing at the time of the next event following them. This method has been previously implemented in the SpiNNaker system under their “deferred event driven model” (Rast et al., 2008; Diehl and Cook, 2014; Galluppi et al., 2015). It is similar to our proposed method in that weight updates are driven by pre-synaptic spikes and causal updates are delayed; however, some important distinctions should be highlighted:

- A neuron's spike history is stored as a bitmap in an array. However, as presented in Appendix A1, using multiple timers is at least as efficient as using a bitmap array, and becomes extremely more efficient for large *T*_*refr*_.
- Acausal updates are not immediately processed and are also deferred to the future, once more pre-synaptic spikes have arrived. This implies that larger arrays are required to store spikes on both sides of the STDP window for post-synaptic neurons. In fact, in their work the post-synaptic bitmap array is three times larger than the pre-synaptic array. In our solution, applying the acausal updates immediately at the onset of pre-synaptic spikes demands that we use timers that must cover only one side (i.e., the longest side) of the STDP window.
- Since the bitmap array is only updated at the onset of new spikes (but not necessarily at the expiration of the pre-synaptic STDP timer) and STDP updates can only take place when an “old” pre-synaptic spike eventually exits the bitmap array, this means that *both* causal and acausal updates rely on frequently firing pre-synaptic neurons. This demands that pre-synaptic spikes arrive at a high enough rate to ensure that the pre-synaptic spike time bitmap array is frequently updated so weight updates are not lost. In their work, the minimum firing rate for pre-synaptic neurons is 10.4 Hz.
- Since multiple pre-synaptic spikes may occur before an “old” pre-synaptic spike eventually exits the bitmap array, this implies that the weights being used for updating post-synaptic neuron variables at each pre-synaptic spike event could (or most likely will) be an “old” set of weights since the causal and acausal updates have been deferred. Therefore, though qualitatively similar, a quantitative equivalence with the original STDP algorithm will probably not occur.

## 5. Conclusions

There are multiple forms of organizing data structures for storing synaptic weights. Among these different memory arrangements, pointer-based models are capable of data compression by storing only the existent connections in the network. In pointer-based models, weights are stored, in a high-level sense, as lists of post-synaptic addresses and weights, where the pointer to the list is defined by the pre-synaptic neuron address. Biologically relevant neural networks are typically unstructured and sparsely connected, making pointer-based architectures particularly efficient at storing these network topologies. In this work, we studied the storage costs (in bits) of each data structure and identified the most efficient based on network parameters (e.g., network size and weight bit-length) and connectivity density.

For the different data structures, we analyzed the computational complexity (in number of memory accesses) of obtaining synaptic address and weight when accessing the tables in forward and reverse directions. Though efficient in terms of storage for a wide range of connectivity density values, pointer-based models natively present only forward connectivity access, making them inefficient when implementing spike-time-based local learning rules such as STDP—which requires both forward (pre-to-post) and reverse (post-to-pre) connectivity access. Therefore, we devised a novel means of efficiently implementing STDP by forward-only synaptic connectivity access, benefiting from the reduced memory storage property of pointer-based data structures. In the traditional STDP algorithm, causal updates are performed at the onset of post-synaptic spikes, demanding reverse access at this instant. Our proposed method operates by delaying the causal weight updates until the instant of expiration of the pre-synaptic STDP timer. With this, forward access is performed for both causal and acausal updates, driven by pre-synaptic events.

Natural drawbacks arise when delaying the causal updates, particularly with respect to high-firing post-synaptic neurons. All the drawbacks can be addressed by a very simple rule: the number of STDP timers for each neuron should be equal to the number of spikes which can occur inside the STDP learning window. This rule can be obtained by using multiple timers when *T*_*refr*_ < *T*_*stdp*_, with each timer lasting *T*_*refr*_ time steps. Using this strategy results in the possibility of implementing nearest-neighbor and all-to-all temporal spike interaction. Additionally, by extending the number of timers, the more complex triplet-based temporal interaction can also be deployed.

Lastly, besides the comparison of storage and access costs and efficiencies for each data structure, we devised a budget efficiency figure of merit for a trade-off analysis of the benefits of each model depending on application requirements and storage and access budget. In sum, we feel our work is unique in that it presents a methodology for identifying the optimal memory arrangement solution based on system requirements and network topology, including also the cost of memory access, and supplying the first viable and exact solution for implementing STDP learning in systems organized with either crossbar arrays or forward-only connectivity tables.

## Author Contributions

BP and GC developed the main part of the work, including the algorithms, simulations, analyses, and results. All authors contributed to the manuscript.

### Conflict of Interest Statement

SP and CA were employed by company Intel Corporation. SS was employed by company aiCTX. The remaining authors declare that the research was conducted in the absence of any commercial or financial relationships that could be construed as a potential conflict of interest.
