# Supplementary material for: Memory-Efficient Synaptic Connectivity for Spike-Timing- Dependent Plasticity
Source: Front Neurosci. 2019 Apr 24;13:357. doi: 10.3389/fnins.2019.00357 (PMC6499189; doi:10.3389/fnins.2019.00357)
Supplement: Supplementary file 1 [file Data_Sheet_1.pdf]

APPENDICES

A1 STORING SPIKE TIMES: BITMAP VS. MULTIPLE TIMERS

Consider a discrete-time system with refractory period of duration T_{refr} and STDP learning window of duration T_{stdp}, both being integer multiples of the system time step, Δt. There are, therefore, two basic options for storing spike history. The first option is to store spikes as a bitmap array, where each position of the array represents a system time step in the past, and spikes which have occurred are represented by 1's. The second option is to store spike times using multiple timers, where each timer counts down from T_{refr} to 0. In this case, whenever a timer expires (reaches zero), it initializes its subsequent timer to value T_{refr}, and the chain of timers propagates in this fashion until the last timer expires.

Let us consider now the two scenarios which may arise in terms of the duration of the refractory period with respect to that of the STDP window:

- $T_{refr} \geq T_{stdp}$: Only one spike can occur inside the STDP window, and thus storing the spike time as a countdown timer requires a single timer of duration $\lceil \log_2(T_{refr} + 1) \rceil$.
- $T_{refr} < T_{stdp}$: Multiple spikes can occur inside the STDP window, requiring $\lfloor T_{stdp}/T_{refr} \rfloor$ timers with duration $(T_{refr} + 1)$, and an additional shorter timer for the remaining time steps in case T_{stdp} is not a multiple of T_{refr}.

To quantitatively assess these scenarios, in Fig. 9A we present results for the difference in costs (in number of bits) required to store the spike times in a system configured with T_{refr} ranging from 1 to 20 and T_{stdp} ranging from 1 to 40. The plot shows that using multiple timers is at least as efficient as using a bitmap, and becomes extremely more efficient for large refractory periods. Figures 9B and 9C show how spikes traverse in memory when using a bitmap and using multiple timers. In the examples, the refractory period is set to $Trefr = 5$ and the STDP window set to $T_{stdp} = 12$. The bitmap uses $\max(T_{refr}, T_{stdp})$ = 12 bits. The multiple timers method requires 2 $\times$ 3-bit timers (to count from T_{refr} to 0) and a single 2-bit timer (to count from 2 to 0) which accounts for the 2 remaining time steps, totaling 8 bits.

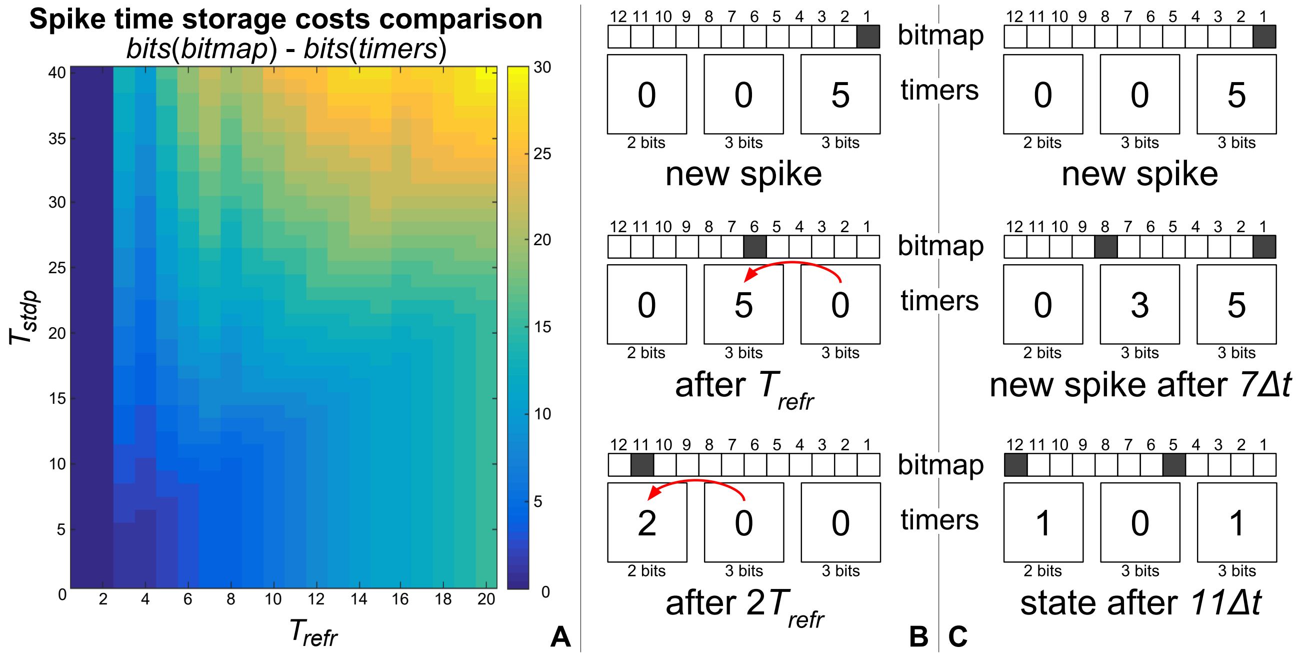

Figure 9. (A) Comparison of spike time storage costs (in number of bits) between using a bitmap and using multiple timers. The heatmap shows that using multiple timers is always at least as efficient as using a bitmap. (B) Example of a single spike traversing through a bitmap and through multiple timers. The refractory period is set to $Trefr = 5$ and the STDP window set to $T_{stdp} = 12$. The bitmap requires 12 bits, while the alternative uses 3 timers totaling 8 bits ($2 \times$ 3-bit + $1 \times$ 2-bit). As a timer expires (reaches zero), it initializes its neighboring timer to T_{refr}; except for the last timer, which accounts for 2 time steps and is, thus, initialized to 2. (C) Example of two spikes traversing through a bitmap and through multiple timers (same parameters as in (B)).
